# Comparative Chloroplast Genomics at Low Taxonomic Levels: A Case Study Using *Amphilophium* (Bignonieae, Bignoniaceae)

**DOI:** 10.3389/fpls.2019.00796

**Published:** 2019-06-19

**Authors:** Verônica A. Thode, Lúcia G. Lohmann

**Affiliations:** Departamento de Botânica, Instituto de Biociências, Universidade de São Paulo, São Paulo, Brazil

**Keywords:** chloroplast genome, comparative genomics, neotropical lianas, NGS, plastome, species-level plastome evolution

## Abstract

Chloroplast (cp) genome organization, gene order, and content have long been considered conserved among land plants. Despite that, the generation of thousands of complete plastomes through next-generation sequencing (NGS) has challenged their conserved nature. In this study, we analyze 11 new complete plastomes of *Amphilophium* (Bignonieae, Bignoniaceae), a diverse genus of Neotropical lianas, and that of *Anemopaegma prostratum*. We explored the structure and content of the assembled plastomes and performed comparative analyses within *Amphilophium* and among other plastomes available for Bignoniaceae. The overall gene content and orientation of plastomes is similar in all species studied. Plastomes are not conserved among *Amphilophium*, showing significant differences in length (155,262–164,786 bp), number of genes duplicated in the IRs (eight, 18, or 19), and location of the SC/IR boundaries (i.e., LSC/IRa junction between *rps19* and *rpl2* genes, within *petD*, or within *petB*). Length differences reflect expansions of the IRs and contractions of the LSC regions. The plastome of *A. prostratum* is 168,172 bp, includes 19 duplicated genes, and has the LSC/IRa boundary located within the *petB* gene. *Amphilophium* plastomes show high nucleotide diversity, with many hypervariable regions, and 16 genes with signatures of positive selection. Multiple SSRs and repeat regions were identified for *Amphilophium* and *Anemopaegma prostratum*. The differences in structure detected within *Amphilophium* plastomes in terms of LSC/IR and IR/SSC boundaries, number of duplicated genes, and genome sizes are mostly shared between taxa that belong to the same clade. Our results bring new insights into the evolution of plastomes at low taxonomic levels.

## Introduction

Chloroplasts are photosynthetic organelles that have an important role in plant carbon fixation, as well as in the biosynthesis of starch, fatty acids, amino acids, and pigments (Jansen and Ruhlman, 2012; Zhao et al., 2015; Daniell et al., 2016). In angiosperms, the chloroplast genome (plastome) generally has a circular structure that ranges from 120 to 180 kb in size and contains a quadripartite structure, composed of two Inverted Repeat (IR) regions, a Large Single Copy (LSC), and a Small Single Copy (SSC) region (Palmer, 1985; Green, 2011). While plastome organization, gene order, and content has been thought to be conserved among land plants (Odintsova and Yurina, 2003; Wicke et al., 2011; Cai et al., 2015; Smith and Keeling, 2015; Reginato et al., 2016), distinct patterns, rearrangements, differences in structure, size, gene content and order have been documented (Chumley et al., 2006; Haberle et al., 2008; Guisinger et al., 2011; Weng et al., 2014; Firetti et al., 2017; Fonseca and Lohmann, 2017). Furthermore, expansions and contractions of IRs with different orders of magnitude have occurred multiple times during land plant evolution (Zhu et al., 2016; Park S. et al., 2018). These shifts result in gene gains or losses attributed to the transfer of genes from SC regions into the IRs or otherwise, leading to plastome size variation among plant lineages (Goulding et al., 1996; Chumley et al., 2006; Raubeson et al., 2007; Wang et al., 2008; Dong et al., 2013; Sun et al., 2013; Zhu et al., 2016; Firetti et al., 2017).

The lianescent genus *Amphilophium* Kunth emend L.G. Lohmann includes 47 species and represents the third largest genus of the Neotropical tribe Bignonieae (Bignoniaceae, Lamiales) (Lohmann and Taylor, 2014). Species of *Amphilophium* occur from Mexico and the Antilles to northern Argentina, southern Brazil, and Uruguay, where they grow in wet and dry forests, or are restricted to savannas or the Amazonian “*campinas”* (Lohmann and Taylor, 2014). Species of the genus have attractive flowers and interesting fruit morphology, being commonly cultivated through South-Western United States (Lohmann, in review), Latin America, and Asia (Pool, 2007a,b). Corolla shape and fruit morphology can be highly variable (Alcantara and Lohmann, 2010). The first molecular phylogenetic studies to sample *Amphilophium* were based on the plastid gene *ndh*F and the nuclear *pep*C and included 11 species (Lohmann, 2006; Lohmann et al., 2013). These studies aimed at re-evaluating generic limits (Lohmann, 2006) and studying broad-scale biogeographical patterns (Lohmann et al., 2013) within the whole tribe Bignonieae. A subsequent phylogenomic study of the genus used sequences of 78 plastid-coding genes of 32 species of *Amphilophium* to reconstruct species-level relationships and the fine-scale biogeographic history of the genus (Thode et al., 2019). Thode et al. (2019) recovered a strongly supported phylogeny of *Amphilophium*, corroborating the monophyly of the genus and its division into five main clades (Lohmann, 2006; Lohmann et al., 2013). These five clades differ morphologically from each other and generally correspond to genera recognized in the past (Gentry, 1973; Pool, 2007a,b, 2009). Despite the existence of phylogenetic and biogeographic information for *Amphilophium* (Lohmann, 2006; Lohmann et al., 2013; Thode et al., 2019), the plastome structure for the members of this genus remains unknown.

The first complete Bignoniaceae plastome reported in the literature was that of *Tanaecium tetragonolobum* (Jacq.) L.G. Lohmann (tribe Bignonieae) (Nazareno et al., 2015). This plastome is 153,776 base pairs (bp) long, with a typical quadripartite structure, including 142 genes. Plastomes of eight *Anemopaegma* species (Firetti et al., 2017), and ten species from the “*Adenocalymma-Neojobertia*” clade (Fonseca and Lohmann, 2017) were published subsequently. Among all Lamiales plastomes published to date, those from *Anemopaegma* are the largest (Firetti et al., 2017). The plastomes of the *Anemopaegma* species range from 167,413 to 168,987 bp and include 141 genes (Firetti et al., 2017). The large size of the *Anemopaegma* plastomes is associated with the large amount of repetitive sequences and expansion of the IRs (Firetti et al., 2017). On the other hand, the plastomes of the “*Adenocalymma-Neojobertia*” clade range from 157,027 to 159,725 bp, and generally include 132 genes, although the *ycf4* gene was lost in two species sampled (Fonseca and Lohmann, 2017). Plastomes of the “*Adenocalymma-Neojobertia*” clade also show a series of genomic translocations (Fonseca and Lohmann, 2017). Apart from the Bignonieae plastomes, the plastome of *Crescentia cujete* L., a member of the Tabebuia alliance (*sensu* Olmstead et al., 2009), was also sequenced (Moreira et al., 2016). This plastome is 154,662 bp in length and includes 132 genes (Moreira et al., 2016).

In this study, we assembled the complete plastomes of 11 species of *Amphilophium* (Bignonieae, Bignoniaceae) representing the breath of the morphological diversity of the genus and the five main clades recovered previously (Lohmann, 2006; Lohmann et al., 2013; Thode et al., 2019), plus that of *Anemopaegma prostratum* DC., an outgroup. This study aims to improve our understanding of plastome characteristics, structural diversity, and evolution within tribe Bignonieae. For that, we: (i) characterized the overall plastome structure; (ii) performed comparative genomic analyses within *Amphilophium*, and among *Amphilophium* and other Bignonieae genera; (iii) documented selection patterns within *Amphilophium* plastid genes; and (iv) identified putative repeated regions.

## Materials and Methods

### Sampling, Sequencing and Annotation

We analyzed 11 plastomes sequenced using an Illumina’s HiSeq 2500 Genome Analyzer (Illumina, San Diego, CA, United States) and assembled by Thode et al. (2019), namely: *A. carolinae* (Lindl.) L.G. Lohmann, *A. chocoensis* (A.H. Gentry) L.G. Lohmann, *A. cuneifolium* (DC.) L.G. Lohmann, *A. dolichoides* (Cham.) L.G. Lohmann, *A. dusenianum* (Kraenzl.) L.G. Lohmann, *A. ecuadorense* A.H. Gentry, *A. gnatophalantum* (A. Rich.) L.G. Lohmann, *A. lactiflorum* (Vahl) L.G. Lohmann, *A. paniculatum* (L.) Kunth, *A. pilosum* Standl., and *A. steyermarkii* (A.H. Gentry) L.G. Lohmann (Table 1). Plastomes assembled for 22 other species (Thode et al., 2019) were not complete and not included here. Furthermore, the plastome of *Anemopaegma prostratum*, another member of tribe Bignonieae, was also assembled in this study and selected as outgroup based on other studies (Lohmann, 2006; Lohmann et al., 2013). More information about DNA preparation, sequencing, and plastome assembly can be found in Thode et al. (2019). The GenBank accession numbers of all 12 plastomes assembled in this study are given in Table 1. In this study, we verified the boundaries between the LSC, the IRs, and the SSC iteratively using the software afin^1^ and by searching the specific motifs from each junction in the original read pool using the UNIX “grep” function for all plastomes assembled. The reads found with the sequences of the junctions between the plastome regions were later assembled in Sequencher 5.3.2 (Genecodes, Ann Arbor, MI, United States). Plastome annotations were performed in Geneious 9.1.5 (Kearse et al., 2012), DOGMA (Wyman et al., 2004), and BLAST (Altschul et al., 1990, 1997), with Open Reading Frames (ORFs) checked manually by searching for the start and stop codons. The graphical representations of each plastome with annotations were created in OGDRAW (Lohse et al., 2013). In addition, the junction sites between the LSC/IRa/SSC/IRb regions with full annotations for the adjacent genes were manually analyzed in Geneious, examined, and plotted in IRscope (Amiryousefi et al., 2018)^2^.

**Table 1:** Taxa, voucher, reference, and GenBank accession numbers of the taxa analyzed in this study.

			GenBank accession
Taxon	Voucher	References	number
*A. carolinae*	M.M. Arbo 9125 (ICN)	This study	MK163625
*A. chocoensis*	M. Monsalve B. 1916 (MO)	This study	MK415793
*A. cuneifolium*	D. Sasaki 2290 (K)	This study	MK415794
*A. dolichoides*	G. Heiden 1769 (SPF)	This study	MK163624
*A. dusenianum*	J. Durigon 582 (ICN)	This study	MK415795
*A. ecuadorense*	D. Rubio 1971 (MO)	This study	MK415796
*A. gnatophalantum*	A.H. Gentry 50829 (MO)	This study	MK135829
*A. lactiflorum*	A.H. Liogier 34305 (MO)	This study	MK163623
*A. paniculatum*	D. Daly 374 (MO)	This study	MK415797
*A. pilosum*	G. Yuncker 5738 (MO)	This study	MK415798
*A. steyermarkii*	J.A. Steyermark 106874 (P)	This study	MK163626
*Anemopaegma prostratum*	J. Durigon 912 (ICN)	This study	MK415799
*Anemopaegma arvense*	F. Firetti 241 (SPF)	Firetti et al., 2017	MF460829
*Adenocalymma peregrinum*	L.H.M. Fonseca 444 (SPF)	Fonseca and Lohmann, 2017	MG008314
*Tanaecium tetragonolobum*	L.G. Lohmann 619 (MO)	Nazareno et al., 2015	KR534325

### Comparative Analyses of Chloroplast Genomes

Comparative analyses were performed between *Amphilophium* and *Anemopaegma prostratum*, as well as between those taxa and other previously published Bignoniaceae plastomes, and within *Amphilophium* only. One copy of the IRs of all plastomes was manually removed in all analyses to avoid data duplication.

To determine synteny and identify possible rearrangements, we compared the *Amphilophium* plastome sequences with those from three other Bignonieae genera [i.e., *Adenocalymma peregrinum* (Miers) L.G. Lohmann (GenBank accession number MG008314, Fonseca and Lohmann, 2017), *Anemopaegma arvense* (Vell.) Stellfeld ex J.F. Souza (GenBank accession number MF460829, Firetti et al., 2017), *Anemopaegma prostratum* (this study), and *Tanaecium tetragonolobum* (Jacq.) L.G. Lohmann (GenBank accession number KR534325, Nazareno et al., 2015)] (Table 1). This analysis was performed in Mauve 2.4.0 (Darling et al., 2010)^3^, with the following settings: progressiveMauve as alignment algorithm, MUSCLE 3.6 (Edgar, 2004) as the internal aligner, with full alignment and minimum locally collinear block (LCB) score automatically calculated. Genomes were not assumed to be collinear.

The 11 *Amphilophium* plastome sequences were aligned in MAFFT 7 (Katoh and Standley, 2013) using the FFT-NS-2 method (Katoh et al., 2002). To identify variable regions and intra-generic variations within the genus, we visualized the alignment using mVISTA (Frazer et al., 2004) in Shuffle-LAGAN mode (Brudno et al., 2003), using the annotated plastome of *A. paniculatum* as reference. The same alignment was used to calculate the nucleotide variability values (π) within *Amphilophium* plastomes. The sliding window analysis was performed in DnaSP 6.10 (Rozas et al., 2017) with step size of 200 bp and window length of 800 bp. We plotted the π values using R (R Development Core Team, 2017).

We estimated the percentage and total number of variable sites across the *Amphilophium* plastomes using MEGA 7 (Kumar et al., 2016). A total of 78 protein-coding genes were extracted from the 11 *Amphilophium* plastomes for all taxa and aligned separately considering codon positions in Geneious, using the translation alignment tool ClustalW plugin (Larkin et al., 2007): i.e., *accD, atpA, B, E, F, H, I, ccsA, cemA, clpP, infA, matK, ndhA, B, C, D, E, F, H, I, J, K, petA, B, D, G, L, N, psaA, B, C, I, J, psbA, B, C, D, E, F, H, I, J, K, L, M, N, T, Z, rbcL, rpl2, 14, 16, 20, 22, 23, 32, 33, 36, rpoA, B, C1, C2, rps2, 3, 4, 7, 8, 11, 12, 14, 15, 16, 18, 19, ycf1, 2, 3*, and *4*. We also estimated the number of variable sites within each of the 78 protein-coding genes with MEGA 7.

### Selection on Plastid Genes

To evaluate the role of selection on the plastid-coding regions, we used the CODEML application in PAML 4.8 (Yang, 2007) performing a Bayesian identification of codon sites under positive selection. This analysis infers the omega values (ω) in codon alignments of protein-coding sequences and tests for positive selection. The omega value measures the ratios of the non-synonymous and synonymous substitution (ω = dN/dS) (Nielsen and Yang, 1998). Sites are considered to be under negative selection (deleterious or purifying selection) when ω < 1; under neutrality (when the substitution does not change the amino acid) when ω = 1; and under positive selection (adaptive selection) when ω > 1. The fixation of advantageous mutations (adaptive evolution) may be related to evolutionary innovations and species divergence. The 78 protein-coding genes (see above) of the 11 *Amphilophium* plastomes and that of *Anemopaegma prostratum* were aligned in Geneious, using the translation alignment tool ClustalW plugin. The CODEML analysis for each gene was performed using as the constraint topology the ML tree from Thode et al. (2019). The terminal and corresponding internal branches of the taxa that were not sampled in this study were removed from the tree in the R package “ape” (Paradis and Schliep, 2018) using the function “drop.tip.” *Anemopaegma prostratum* was designated as outgroup. Parameters were: runmode = 0, seqtype = 1, CodonFreq = 2, and model = 0, and NSsites = 2 (modeling three classes of sites: 0 < = ω < 1, ω = 1, and ω > 1). Results were considered significant when the posterior probability (Pr) >0.95.

### Repeat Analyses

We used MISA (Beier et al., 2017) to identify and locate microsatellites or Simple Sequence Repeats (SSRs; i.e., tandemly arranged repeats of short DNA motifs of 1–6 bp in length) in the plastomes of the *Amphilophium* species and *Anemopaegma prostratum*. The following criteria were used while searching for SSRs: SSR motif length between one and six nucleotides, with a minimum number of repetitions set as 10, 5, and 4 units for mono-, di-, and trinucleotide SSRs, respectively, and three units for each tetra-, penta-, and hexanucleotide SSRs. We used REPuter (Kurtz et al., 2001) to identify forward, palindrome, reverse, and complement repeated elements with a minimum repeat size ≥30 bp and a sequence identity ≥90% (Hamming distance = 3).

## Results

### Assembly and Characteristics of the Chloroplast Genomes

**FIGURE 1:**
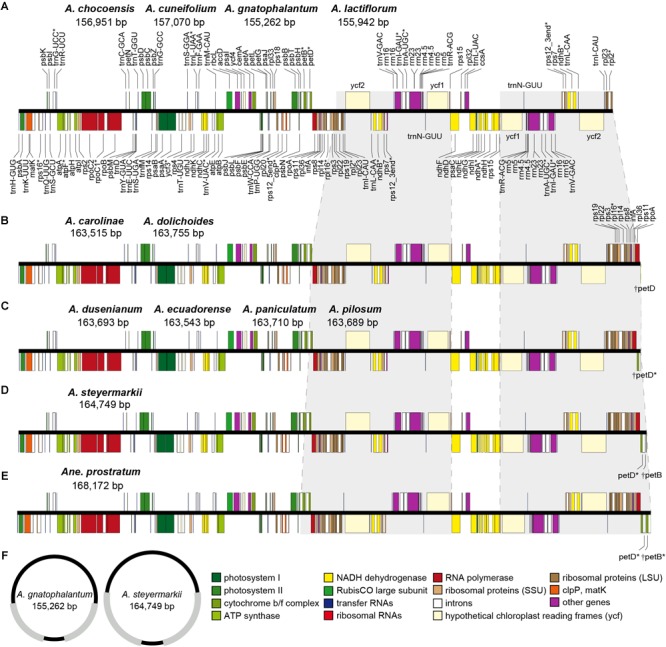
**(A–E)** Gene maps of the plastomes of the *Amphilophium* and *Anemopaegma* species assembled in this study. Gray shading highlights IR regions with IR boundary shifts. Genes drawn below the line are transcribed clockwise, and those drawn above the line are transcribed counterclockwise. Genes belonging to different functional groups are colored according to the legend. Asterisks (^∗^) represent intron-containing genes. **(F)** Representation of the smallest and largest *Amphilophium* plastomes studied. Gray regions correspond to the IRs.

**FIGURE 2:**
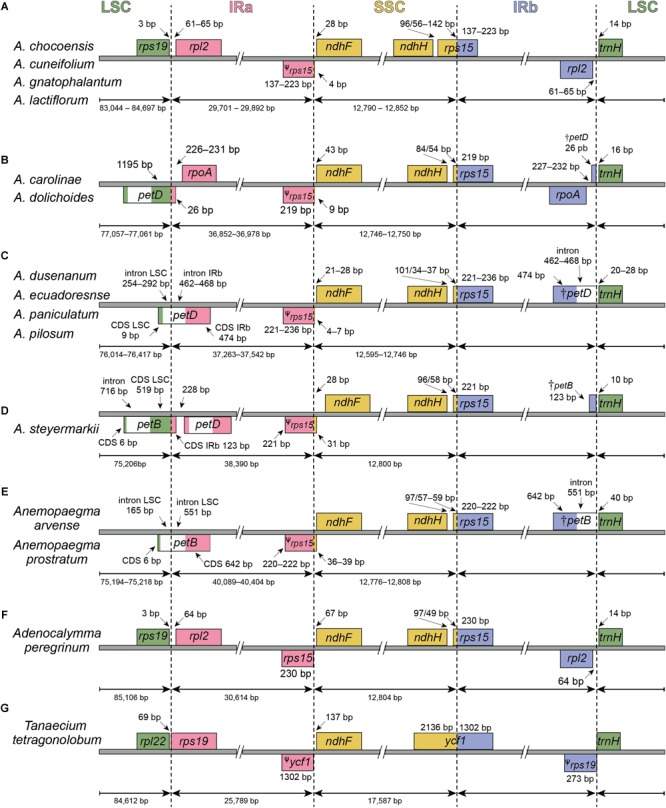
Comparisons of the Large Single Copy (LSC), Inverted Repeat a (IRa), Small Single Copy (SSC), and Inverted Repeat b (IRb) boundaries **(A–D)** within *Amphilophium* and **(E–G)** among four other Bignoniaceae plastomes. Genes shown below are transcribed reversely and those shown above the lines are transcribed forward. Minimum and maximum sizes for the regions and structures of each plastome type that compose the borders are indicated in base pairs (bp).

The eleven *Amphilophium* plastomes range in length from 155,262 (*A. gnatophalantum*) to 164,749 bp (*A. steyermarkii*) (Table 2, Figure 1, and Supplementary Figures S1, S2). A minimum of 8,102,426 paired-end raw reads, and a maximum of 23,885,903 reads, with average read depths between 54.5 and 248x for *A. cuneifolium* and *A. dolichoides* were obtained, respectively (Supplementary Table S1). All plastomes show the typical quadripartite structure of angiosperms, which consists of a LSC, with length between 75,206 (*A. steyermarkii*) and 84,697 bp (*A. chocoensis*); a SSC with length between 12,595 (*A. dusenianum*) and 12,852 bp (*A. chocoensis*); and a pair of IRs with length between 29,701 (*A. chocoensis*) and 38,390 bp (*A. steyermarkii*) (Table 2, Figure 1, 2, and Supplementary Figures S1, S2A). *Anemopaegma prostratum* exhibits the largest plastome assembled in this study, with a total length of 168,172 bp, including a LSC composed by 75,218 bp, a SSC with 12,776 bp, and IRs with 40,089 bp, similar to that of *Anemopaegma arvense* (Firetti et al., 2017; Table 2, Figure 1, 2, and Supplementary Figure 2A). The IR is expanded at the LSC/IRa and IRb/LSC boundaries in some *Amphilophium* species and in *Anemopaegma* relative to *Adenocalymma peregrinum* (Fonseca and Lohmann, 2017) and *Tanaecium tetragonolobum* (Nazareno et al., 2015; Table 2 and Figure 1, 2). The coding regions of the 11 *Amphilophium* plastomes range from 83,262 (*A. chocoensis*) to 88,536 bp (*A. steyermarkii*). The noncoding regions vary from 71,907 (*A. gnatophalantum*) to 76,284 bp (*A. paniculatum*). In *Anemopaegma prostratum* the coding regions are 89,640 bp in length, while the noncoding regions are 78,532 bp (Table 2 and Supplementary Figure S2B). The average GC content is 37.8% for all species studied (Table 2), similar to other Bignoniaceae plastomes sequenced to date (Nazareno et al., 2015; Moreira et al., 2016; Firetti et al., 2017; Fonseca and Lohmann, 2017).

**Table 2:** Summary of the *Amphilophium* and *Anemopaegma* plastomes sequenced.

	Plastome	LSC	IR	SSC	Coding	Noncoding	GC	
	length	length	length	length	regions	regions	content	Unique	Unique	Total	Total	Total	Total
Species	(bp)	(bp)	(bp)	(bp)	(bp)	(bp)	(%)	genes	CDS	CDS	tRNA	rRNA	genes
*A. gnatophalantum*	155,262	83,044	29,714	12,790	83,355	71,907	37.8	113	79	87	37	8	132
*A. lactiflorum*	155,956	83,637	29,754	12,810	83,462	72,494	37.9	113	79	87	37	8	132
*A. chocoensis*	156,951	84,697	29,701	12,852	83,262	73,689	37.9	113	79	87	37	8	132
*A. cuneifolium*	157,070	84,452	29,892	12,834	83,286	73,784	37.9	113	79	87	37	8	132
*A. carolinae*	163,515	77,061	36,852	12,750	88,020	75,495	37.8	113	79	97	37	8	142
*A. dolichoides*	163,755	77,057	36,978	12,746	88,065	75,690	37.8	113	79	97	37	8	142
*A. ecuadorense*	163,543	76,263	37,279	12,722	87,303	76,240	37.8	113	79	97	37	8	142
*A. pilosum*	163,689	76,417	37,263	12,746	88,245	75,444	37.8	113	79	97	37	8	142
*A. dusenianum*	163,693	76,014	37,542	12,595	88,102	75,591	37.7	113	79	97	37	8	142
*A. paniculatum*	163,710	76,228	37,372	12,738	87,426	76,284	37.7	113	79	97	37	8	142
*A. steyermarkii*	164,786	75,206	38,390	12,800	88,536	76,250	37.7	113	79	98	37	8	143
*Ane. prostratum*	168,172	75,218	40,089	12,776	89,640	78,532	37.7	113	79	98	37	8	143

The 12 plastomes assembled here encode 113 unique genes, including 79 protein-coding genes (CDS), 30 tRNA genes, and four rRNA genes (Tables 2, 3 and Supplementary Table S2). The number of duplicated CDS in the IRs varies depending on the degree of IR expansion and contraction of the LSC regions. While some species show eight duplicated CDS in the IRs (i.e., *ndhB, rpl2, rpl23, rps12, rps7, ycf1, ycf2*, and *ycf15*), others show 18 (i.e., the previous eight regions plus *infA, rpl14, rpl16, rpl22, rpl36, rpoA, rps11, rps19, rps3*, and *rps8*), or 19 duplications (the previous 18 regions plus *petD*) (Tables 2–4 and Figure 1). All species include seven tRNA and all four rRNA genes duplicated in the IR regions. The total number of genes ranges from 132 to 143 (Tables 2, 4 and Figure 1). The plastomes assembled in this study include 18 intron-containing genes, of which 15 contain one intron (i.e., *atpF, ndhA, ndhB, petB, petD, rpl2, rpl16, rpoC1, rps16, trnA-UGC, trnG-UCC, trnI-GAU, trnK-UUU, trnL-UAA*, and *trnV-UAC*), while three genes contain two introns (i.e., *cplP, rps12, ycf3*) (Table 3 and Figure 1). The *rps12* gene is trans-spliced, with the 5′ end located in the LSC region and the duplicated 3′ end in the IR regions.

**Table 3:** Genes encoded by the *Amphilophium* species and *Anemopaegma prostratum* plastomes.

Gene Functoin	Gene Type	Gene
Self-replication	∙ Ribossomal RNA genes	*rrn4.5^a^, rrn5^a^, rrn16^a^, rrn23^a^*
	• Transfer RNA genes	*trnA-UGC^∗a^, trnC-GCA, trnD-GUC, trnE-UUC, trnF-GAA, trnfM-CAU, trnG-UCC, trnG-UCC^∗^, trnH-GUG, trnI-CAU, trnI-GAU^∗a^, trnK-UUU^∗^, trnL-CAA^a^, trnL-UAA^∗^, trnL-UAG, trnM-CAU^a^, trnN-GUU^a^, trnP-UGG, trnQ-UUG, trnR-ACG^a^, trnR-UCU, trnS-GCU, trnS-GGA, trnS-UGA, trnT-GGU, trnT-UGU, trnV-GAC^a^, trnV-UAC^∗^, trnW-CCA, trnY-GUA*
	• Small ribosomal subunit	*rps2, rps3^b^, rps4, rps7^a^, rps8^b^, rps11^b^, rps12^∗∗a^, rps14, rps15^a^, rps16^∗^, rps18, rps19^b^*
	• Large ribosomal subunit	*rpl2^∗a^, rpl14^b^, rpl16^∗ b^, rpl20, rpl22^b^, rpl23^a^, rpl32, rpl33, rpl36^b^*
	• RNA polymerase subunits	*rpoA^b^, rpoB, rpoC1^∗^, rpoC2*
Photosynthesis	• Photosystem I	*psaA, psaB, psaC, psaI, psaJ^∗^*
	• Assembly/stability of photosystem I	*ycf3^∗^, ycf4*
	• Photosystem I	*psbA, psbB, psbC, psbD, psbE, psbF, psbH, psbI, psbJ, psbK, psbL, psbM, psbN, psbT, psbZ*
	• NADH dehydrogenase	*ndhA^∗^, ndhB^∗a^, ndhC, ndhD, ndhE, ndhF, ndhG, ndhH, ndhI, ndhJ, ndhK*
	• Cytochrome b/f complex	*petA, petB^∗^, petD^∗ c^, petG, petL, petN*
	• ATP synthase	*atpA, atpB, atpE, atpF^∗^, atpH, atpI*
	• Rubisco	*rbcL*
Other genes	• Translational initiator factor	*infA^b^*
	• Maturase	*matK*
	• Protease	*clpP^∗∗^*
	• Envelope membrane protein	*cemA*
	• Subunit of Acetil-CoA-carboxylase	*accD*
	• c-type cytochrome synthesis	*ccsA*
Pseudogenes in some species		ψ*petB*,ψ*petD*,ψ*rps15*
Unknown function	• Hypotetical chloroplast reading frames	*ycf1^a^, ycf2^a^*

*^∗^Genes with one intron. ^∗∗^Genes with two introns. ^*a*^Genes duplicated in all taxa. ^*b*^Genes duplicated in *A. carolinae, A. dolichoides, A. dusenianum, A. ecuadorense, A. paniculatum, A. pilosum, A. steyermarkii*, and *Anemopaegma prostratum.*^*c*^Gene duplicated in *A. steyermarkii* and Anemopaegma prostratum.*

**Table 4:** Comparisons of the junctions between the Large Single Copy (LSC) and Inverted Repeat a (IRa) and the Inverted Repeat b (IRb) and Small Single Copy (SSC) and number of duplicated protein-coding genes (CDS) in the IRs within *Amphilophium* and among four other Bignoniaceae plastomes.

	LSC/IRa	IRb/LSC	Duplicated
Species	boundary	boundary	CDS
*A. gnatophalantum*	*rps19* and *rpl2*	*rpl2* and the	8
*A. lactiflorum*		*trnH-GUG*	
*A. chocoensis*			
*A. cuneifolium*			

*A. carolinae*	within *petD* exon II	†*petD* (26 bp) and	18
*A. dolichoides*		*trnH-GUG*	

*A. ecuadorense*	within *petD* intron	†*petD* (936–942 bp)	18
*A. pilosum*		and *trnH-GUG*	
*A. dusenianum*			
*A. paniculatum*			

*A. steyermarkii*	within *petB* exon II	†*petB* (123 bp) and *trnH-GUG*	19

*Ane. prostratum*	within *petB* intron	†*petB* (1,193 bp)	19
*Ane. arvense*		and *trnH-GUG*	

*Ade. peregrinum*	*rps19* and *rpl2*	*rpl2* and the *trnH-GUG*	8

*Tan. tetragonolobum*	*rps22* and *rps19*	ψ*rps19* and *trnH-GUG*	7

According to the IRs/LSC boundaries and the number of duplicated CDS in the IRs, four main plastome patterns were detected within *Amphilophium* (Tables 2, 4 and Figure 1, 2). The plastomes of *A. chocoensis, A. cuneifolium, A. gnatophalantum*, and *A. lactifluorum* have the LSC/IRa boundary between the *rps19* and *rpl2* genes with eight completely duplicated CDS in the IRs (Table 4 and Figure 2A). The plastomes of *A. carolinae, A. dolichoides, A. dusenianum, A. ecuadorense, A. paniculatum*, and *A. pilosum* have the LSC/IRa boundary within the *petD* gene with 18 duplicated CDS. The IR expansion includes a C-terminal portion of *petD* generating a truncated (†) *petD* fragment in IRb. These expansions result in a smaller LSC containing the N-terminal portion of *petD* (Table 4 and Figure 2B,C). The †*petD* in the IRb of *A. carolinae* and *A. dolichoides* have only 26 bp (Figure 2B), whereas in *A. dusenianum, A. ecuadorense, A. paniculatum*, and *A. pilosum* the †*petD* have 936–942 bp (Figure 2C). The plastomes of *A. steyermarkii* and *Anemopaegma prostratum* have the LSC/IRa boundary within the *petB* gene with 19 duplicated CDS. The IR expansion in these two taxa includes a C-terminal portion of *petB* generating a †*petB* fragment in IRb. The LSC in these species are the smallest among the analyzed plastomes and contain the N-terminal portion of *petB*. The †*petB* in *A. steyermarkii* IRb has only 123 bp (Figure 2D), whereas in *Anemopaegma prostratum* it has 1,193 bp (Table 4 and Figure 2E).

In all *Amphilophium* studied, one copy of the duplicated *rps15* is a pseudogene (ψ) that is 141–240 bp long and is located within the boundary between IRa/SSC, while the functional *rps15* gene is 270–279 bp long and located within the SSC/IRb border. In *Anemopaegma prostratum*, the ψ*rps15* is 261 bp long, while the *rps15* gene is 279 bp (Figure 2). The IRb/LSC junction in *A. chocoensis, A. cuneifolium, A. gnatophalantum*, and *A. lactiflorum* is between *rpl2* and the *trnH-GUG* genes (Figure 2A); in *A. carolinae, A. dolichoides, A. dusenianum, A. ecuadorense, A. paniculatum*, and *A. pilosum* it is between †*petD* and *trnH-GUG* (Figure 2B,C); and in *A. steyermarkii, Anemopaegma prostratum*, and *Anemopaegma arvense* (Firetti et al., 2017) it is between †*petB* and *trnH-GUG* (Table 4 and Figure 2D,E). The structure found in the IRa/SSC/IRb borders of the *A. steyermarkii* and *Anemopaegma prostratum* is similar to that found in *Anemopaegma arvense* (Firetti et al., 2017; Figure 2D,E), and in the plastomes of seven other *Anemopaegma* species (Firetti et al., 2017). In *Adenocalymma peregrinum* (Fonseca and Lohmann, 2017), all boundaries are similar to those found in *A. chocoensis, A. cuneifolium, A. gnatophalantum*, and *A. lactiflorum* (Figure 2A,F). The boundaries between all regions are different in the plastome of *Tanaecium tetragonolobum* (Nazareno et al., 2015; Figure 2G) when compared to those from *Amphilophium, Adenocalymma*, and *Anemopaegma* (Figure 2). In *T. tetragonolobum*, the LSC/IRa boundary is located between the *rpl22* and *rps19* genes, while the IRa/SSC border is located between ψ*ycf1* and the *ndhF* gene, and the SSC/IRb border is within the *ycf1* gene (Nazareno et al., 2015; Table 4 and Figure 2G). The plastomes of *Amphilophium, Adenocalymma*, and *Anemopaegma* include an entire duplication of the *ycf1* gene in the IRs (Firetti et al., 2017; Fonseca and Lohmann, 2017; Figure 1, 2).

### Identification of Variable Regions

The structural analysis performed in Mauve retrieve five synteny blocks (Supplementary Figure S3). *Amphilophium* and *Adenocalymma peregrinum* plastomes (Fonseca and Lohmann, 2017) show the same structure and linear order and are similar to those observed in *Anemopaegma arvense* (Firetti et al., 2017), *Anemopaegma prostratum*, and *Tanaecium tetragonolobum* (Nazareno et al., 2015), except for two local changes. The first is a large inversion of approximately 8 kb, located in the IR regions of both *Anemopaegma* plastomes, comprising the genes *rpl23, trnL-CAA, ycf2*, and *trnI-AAU* (Supplementary Figure S3: yellow block). The second is a smaller inversion (∼1,800 bp) observed within the *ycf1* gene in the plastome of *Tanaecium tetragonolobum* (Supplementary Figure S3: blue block). No major inversions are found within the *Amphilophium* and *Adenocalymma peregrinum* plastomes (Supplementary Figure S3).

**FIGURE 3:**
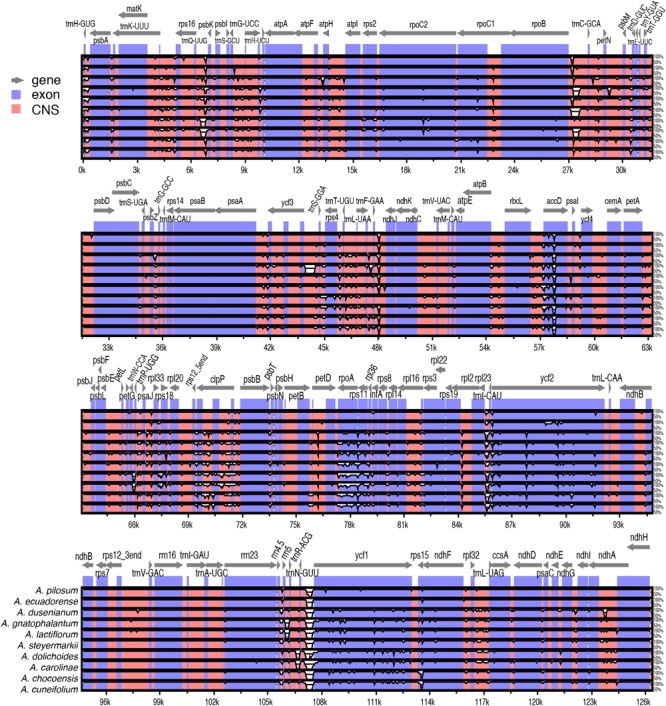
Comparison of the assembled *Amphilophium* plastomes using mVISTA. Complete plastomes of *Amphilophium* species are compared using *A. paniculatum* as reference. Blue blocks indicate conserved genes, while red blocks indicate conserved noncoding sequences (CNS). White blocks represent regions with sequence variation among the 11 *Amphilophium* species. Gray arrows indicate the direction of gene transcription.

Pairwise comparison of divergent regions within the 11 *Amphilophium* plastomes was performed using mVISTA, with *A. paniculatum* as a reference (Figure 3). Overall, the alignment reveals intra-generic sequence divergence across the plastomes, suggesting that plastomes are not conserved. Noncoding regions are generally more divergent than coding regions. Ten noncoding regions show high divergence among the *Amphilophium* plastomes: nine intergenic spacers, *trnH-GUG/psbA, trnQ-UUG/psbK, rpoB/trnC-GCA, trnF-GAA/ndhJ, psaJ/rpl33, trnI-CAU/ycf2, trnN-GUU/ycf1, ndhF/rpl32, rpl32/trnL-UAG*, and *clpP* introns. Seven coding regions exhibit high divergence, *accD, clpP, petD, rpoA, rps11, ycf2*, and *ycf1*, among the studies plastomes (Figure 3).

**FIGURE 4:**
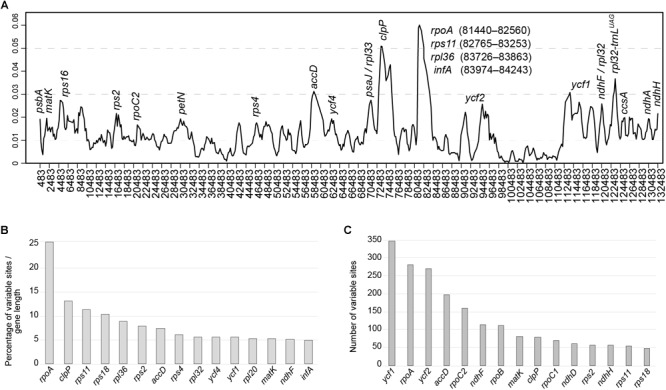
**(A)** Sliding window analysis of the complete plastomes of 11 *Amphilophium* species (window length: 800 bp, step size: 200 bp). *X*-axis, position of the midpoint of each window; *Y*-axis, nucleotide diversity (π) of each window. **(B,C)** Fifteen most variable protein-coding genes within the assembled *Amphilophium* plastomes. **(B)** Percentage of variable sites according to gene length. **(C)** Number of variable sites per gene.

To elucidate levels of diversity at the sequence level, we calculated the nucleotide variability (π) values within the 11 *Amphilophium* plastomes (Figure 4A). The π values within 800 bp across the plastomes range from 0 to 0.06292, with mean value of 0.01224, indicating that these sequences are highly variable. We identified three hypervariable sites with π > 0.05, which are *rpoA, clpP*, and *rps11*; five with π between 0.049 and 0.03, which are *accD, rps12_5end/clpP, petD, trnN-GUU/ycf1*, and *rpl32/trnL-UAG*; and five with π > 0.025, which are *rpl36, ycf1, rps18, matK/rps16*, and *ycf2* (Figure 4A).

In multiple alignments of the *Amphilophium* plastomes assembled here, the noncoding regions are more variable (i.e., 5.12% of the intergenic regions or 3,221 variable sites from 62,946 bp and 4.25% of the introns or 756 variable sites from 17,804 bp) than the coding regions (4.06% of the protein-coding genes or 2,868 variable sites from 70,554 bp). Among the 78 protein-coding genes, the 15 genes with the highest percentage of variable sites are: *rpoA* (25.9%), *clpP* (13%), *rps11* (11.2%); *rps18* (10.3%), *rpl36* (8.8%), *rps2* (7.8%), *accD* (7.4%), *rps4* (6%), *rpl32* (7.8%), *ycf4* (5.5%), *ycf1* (5.5%), *rpl20* (5.3%), *matK* (5.2%), *ndhF* (5.1%), and *infA* (5%) (Figure 4B and Supplementary Table S2). In terms of absolute numbers, the 15 genes with the highest number of variable sites are: *ycf1* (346), *rpoA* (334), *ycf2* (270), *accD* (198), *rpoC2* (159), *ndhF* (114), *rpoB* (112), *matK* (80), *clpP* (78), *rpoC1* (70), *rps2* (56), *ndhH* (56), *rps11* (55), and *rps18* (47) (Figure 4C and Supplementary Table S2).

### Selection on Plastid Genes

The analyses conducted in CODEML to investigate the selection pressure on the 78 protein-coding genes within *Amphilophium* plastomes, indicated that 16 genes are under positive selection (adaptive selection), when ω > 1 with Pr > 0.95. These genes are: *ycf1* (31 sites), *ycf2* (25 sites), *rpoA* (15 sites), *accD* (12 sites), *rps18* and *rps7* (11 sites), *ycf4* (8 sites), *clpP* and *rbcL* (5 sites each), *rpoC1* and *rps2* (4 sites each), *rpoC2* and *infA* (2 sites each), *atpA, rps8* and *rps16* (1 site each). Out of the 23,528 codon sites (corresponding to 70,554 bp) of the 78 protein-coding genes, 138 are under positive selection (ω > 1, Pr > 0.95) (Supplementary Table S2). In other genes, sites are probably under neutrality (substitution does not lead to amino acid change, when ω = 1), or sites are under purifying selection (deleterious or constraining selection, when ω < 1).

### SSR and Tandem Repeat Analyses

**FIGURE 5:**
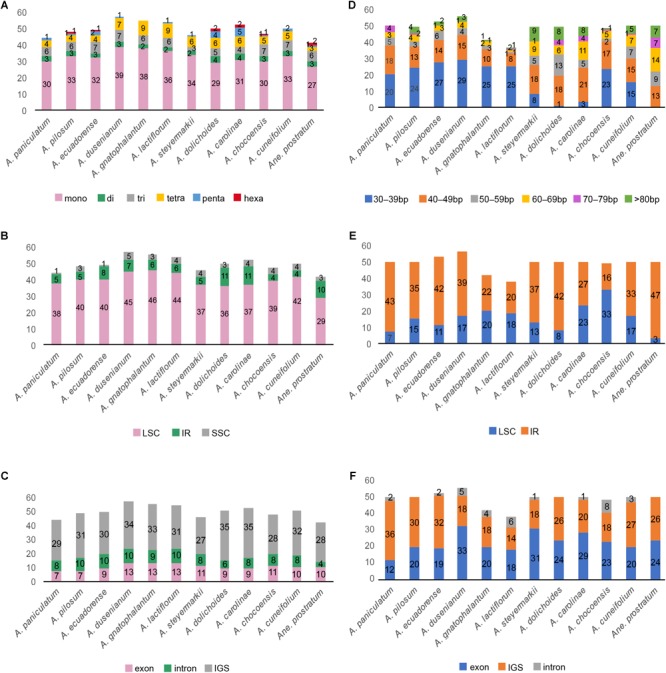
**(A–C)** Distribution of SSRs in the *Amphilophium* and *Anemopaegma prostratum* plastomes. **(A)** Distribution of SSR types. **(B)** Number of SSRs per genomic regions. **(C)** Distribution of SSRs in exon, intergenic spacer (IGS), and intron regions. **(D–F)** Analysis of tandem repeats in the *Amphilophium* and *Anemopaegma prostratum* plastomes. **(D)** Distribution and length of tandem repeats. **(E)** Distribution of tandem repeats in genomic regions. **(F)** Distribution of tandem repeats in exon, intergenic spacer (IGS), and intron regions.

We screened and identified six kinds of repeat patterns using MISA. In *Amphilophium* plastomes, the total number of SSRs range from 44 (*A. paniculatum*) to 57 SSRs (*A. dusenianum*), while 42 SSRs are recovered in *Anemopaegma prostratum* (Figure 5). The most abundant SSRs are A or T mononucleotide repeats, which account for 54–69.6% of the total SSRs; G or C repeats, on the other hand, are rare (Figure 5A and Supplementary Table S3). The total number of SSR motifs in *Amphilophium* is as follows: 29–39 (58–74%) mono-, 2–4 (3.6–8%) di-, 3–7 (6.5–15%) tri-, 4–9 (7–17%) tetra-, 0–5 (0–9.6%) penta-, and 0–2 (0–4.8%) hexanucleotides (Figure 5A and Supplementary Table S3). Furthermore, most of the SSRs in the *Amphilophium* species are located in the LSC region and range between 71.2 and 86.4%. In *Amphilophium*, the IR regions include between 8.5 and 22% of the SSRs, while the SSC region include between 2 and 8.8% (Figure 5B and Supplementary Table S3). SSRs are found mainly in intergenic regions. The plastomes of the *Amphilophium* species contain between 57.4 and 82% of the SSRs in the intergenic spacers, between 14.6 and 24% in the coding regions, and between 12 and 20.8% in the introns (Figure 5C and Supplementary Table S3). In *Anemopaegma prostratum*, 69% of the SSRs are located in the LSC, 23.8% in the IRs, and 7.1% in the SSC region. Of the total number of SSRs found in *A. prostratum*, 66.7% are in the intergenic regions, 23.8% in the coding regions, and 9.5% in the exons (Figure 5B,C and Supplementary Table S3).

We also used REPuter to identify the tandem repeat sequences of ≥30 bp of the *Amphilophium* and *Anemopaegma prostratum* plastomes. The total number of repeats in *Amphilophium* range between 38 (*A. lactiflorum*) and 56 (*A. dusenianum*), all located in the LSC and IR regions, with maximum sizes ranging from 50 to 150 bp (Figure 5D,E and Supplementary Tables S4, S5). The *Amphilophium* plastomes contain between 33 and 50 forward repeats, and 1 to 6 palindrome repeats, with reverse repeats being rare, ranging from 0 to 3 (Supplementary Table S4). In most *Amphilophium* plastomes, repeats with 30–39 bp are the most common, except in *A. carolinae, A. dolichoides*, and *A. steyermarkii*, all of which have a large number of repeats ranging from 40 to 49 bp (Figure 5D and Supplementary Table S4). These repeats are found predominantly in intergenic regions (14–36 bp) and exons (12–33 bp), with a few repeats located in the introns (0–8 bp) (Figure 5F and Supplementary Table S4). In *Anemopaegma prostratum* plastomes, the total number of repeats is 50, three of which are located in the LSC and 47 located in the IR regions; 24 are located in the intergenic regions and 26 in the exons; 49 are forward repeats and one palindrome with a maximum size of 165 bp. Different from the *Amphilophium* plastomes, most of the repeats in *A. prostratum* range between 60 and 69 bp (Figure 5D–F and Supplementary Tables S4, S5). The locations of the repetitive sequences vary among *Amphilophium* species, although some regions show repeats on all 11 species (e.g., *accD, rbcL*/*accD, ycf1*, and *ycf2*), while some locations show repeats on most species (e.g., *rps12/trnV-GAC, trnN-GUU/ycf1, ycf3, psbT/psbN, rps11, rpl23/trnI-CAU*) (Supplementary Table S5).

## Discussion

### Plastome Features

In this study, we assembled 11 complete plastomes of *Amphilophium* species and the plastome of *Anemopaegma prostratum*, another species from tribe Bignonieae. The organization of *Amphilophium* plastomes is similar among the species studied and other angiosperm plastomes. *Amphilophium* plastomes show expansions of the IRs and contractions on the LSC in some species. The overall genomic structure among *Amphilophium* plastomes is not conserved though, including differences in length, boundaries between the SC/IR regions, number of duplicated genes in the IRs, and total length (Tables 2, 4 and Figure 1–4). We detected a difference of nearly 9.5 kb between the smallest (*A. gnatophalantum*) and largest (*A. steyermarkii*) genomes, respectively (Table 2 and Figure 2F). Expansions of the IRs of *ca.* 8.7 kb and LSC contractions of *ca.* 9.5 kb are observed (Table 2, Figure 1, 2 and Supplementary Figure S1). The *Anemopaegma prostratum* plastome also shows an IR expansion and a LSC contraction, similar to the plastomes of eight other *Anemopaegma* species sequenced in a previous study (Firetti et al., 2017). Nonetheless, the IR expansion of *Anemopaegma* plastomes is even larger than those found in *Amphilophium* plastomes, with a *ca.* 10 kb expansion, when the IRs of *A. gnatophalantum* and *Anemopaegma prostratum* are compared. On the other hand, the LSC in the *Anemopaegma prostratum* plastome is *ca.* 9.4 kb smaller than that of *A. chocoensis* (Table 2). The SSC show a small variation in size within *Amphilophium* plastomes, with a difference of 257 bp between the smallest (*A. dusenianum*) and largest regions (*A. chocoensis*) (Table 2 and Figure 1, 2). According to the IR expansion toward the LSC, the *Amphilophium* plastomes exhibit different junctions between regions (i.e., between *rps19* and *rpl2*, within *petD*, and within *petB*), as well as a different number of completely duplicated protein-coding genes (i.e., eight, 18, or 19). Besides the expansion of the IRs and differences in the boundaries of the regions within the *Amphilophium* plastomes, no rearrangements or major inversions are detected. An inversion of ∼8 kb that includes the genes *rpl23, trnL-CAA, ycf2*, and *trnI-AAU* is observed in *Anemopaegma prostratum* and in the plastomes of other eight *Anemopaegma* species. However, these inversions were not observed in any other Lamiales (Firetti et al., 2017; Supplementary Figure S3). The boundary positions observed in *Anemopaegma prostratum* were conserved among eight other *Anemopaegma* plastomes (Firetti et al., 2017). The *Anemopaegma* plastomes are the largest described to date for Lamiales, with 19 completely duplicated CDS in the IRs (Firetti et al., 2017). PCR amplifications were performed to check the boundary positions and the inversion of the *ycf2* gene in *Anemopaegma* (Firetti et al., 2017).

The IR/SC boundaries are conserved in ten plastomes of the “*Adenocalymma-Neojobertia*” clade (Fonseca and Lohmann, 2017). Despite that, the genome structure is quite variable within the “*Adenocalymma-Neojobertia*” clade, with rearrangements in the LSC and IR regions and a complete loss of the *ycf4* gene in two species (Fonseca and Lohmann, 2017). Furthermore, plastomes of the “*Adenocalymma-Neojobertia*” clade show eight duplicated CDS in the IRs (Fonseca and Lohmann, 2017). All the boundaries between plastome regions of *Tanaecium tetragonolobum* (Nazareno et al., 2015) and *Crescentia cujete* (Moreira et al., 2016) are located in positions that are different from those of *Amphilophium, Adenocalymma*, and *Anemopaegma*. In these two species, the LSC/IRb boundary is located between the *rpl22* and *rps19* genes, the IRb/SSC border is located between the ψ*ycf1* and the *ndhF* gene, and the SSC/IRa border is located within the *ycf1* gene (Nazareno et al., 2015; Moreira et al., 2016; Figure 2). The plastomes of these two species also show a partial duplication of the *ycf1* (ψ*ycf1*) and a duplication of the complete copy of the *rps15* gene. Differently, the plastomes of *Amphilophium, Adenocalymma*, and *Anemopaegma* show a complete duplication of the *ycf1* gene as well as a partial duplication of the *ycf15* in the IRs (Firetti et al., 2017; Fonseca and Lohmann, 2017; Figure 1, 2). Part of the *ycf1* and *ycf15* genes are included in the SSC region in other angiosperm groups (Dugas et al., 2015). The shift of the IRs/SSC junctions in *Amphilophium, Anemopaegma*, and the “*Adenocalymma-Neojobertia*” clade result in the expansion of the IRs and contraction of the SSC (Firetti et al., 2017; Fonseca and Lohmann, 2017). The expansion of the IRs toward the SSC has also been reported in *Pelargonium* (Chumley et al., 2006), members of Apiales (Downie and Jansen, 2015), in some Leguminosae genera (Dugas et al., 2015), and in *Lamprocapnos spectabilis* (Papaveraceae) (Park S. et al., 2018). Multiple instances of IR expansion and/or contraction occurred during land plant evolution, with movement of entire genes from the SC regions into the IR or vice-versa (Zhu et al., 2016). The terminal IR gene adjacent to the SSC region is usually more conserved across land plants, however the IR/LSC boundary has changed more dynamically during the evolution of plant lineages (Raubeson et al., 2007; Wang et al., 2008; Dong et al., 2013; Zhu et al., 2016). While most shifts are small, others have expanded or contracted the IR by several kb, resulting in gene gains or losses as a consequence of the relocation of genes into or out of the IR (Goulding et al., 1996; Chumley et al., 2006; Wang et al., 2008; Sun et al., 2013; Zhu et al., 2016; Firetti et al., 2017; Park S. et al., 2018). Notable examples of size variation in the IRs due to boundary shifts are found, for example, in *Monsonia speciosa* (7 kb) (Guisinger et al., 2011), *Lamprocapnos spectabilis* (51 kb) (Park S. et al., 2018), and *Pelargonium transvaalense* (88 kb) (Chumley et al., 2006), though the angiosperm IR is typically 25 kb (Park S. et al., 2018). IR expansions and contractions often result in variation of genome size among different plant groups and are important for plastome evolution (Kim and Lee, 2005; Wang et al., 2008; Asaf et al., 2016; Dong et al., 2016; Yang et al., 2016; Zhang et al., 2016; Zhu et al., 2016; Xu et al., 2017; Li and Zheng, 2018).

The different patterns observed in the *Amphilophium* plastomes in terms of LSC/IR and IR/SSC boundaries, number of duplicated genes, and genome sizes are mostly shared among taxa that belong to the same clade (Thode et al., 2019). *Anemopaegma*, used here as outgroup, showed a plastome structure that is similar to that found in *A. steyermarkii* (Figure 1, 2). Nonetheless, *Anemopaegma* is not necessarily the closest relative of *Amphilophium*, as the genus is sister to a clade comprising *Anemopaegma* Mart. ex Meisn., *Bignonia* L., *Mansoa* DC, and *Pyrostegia* C. Presl (Lohmann, 2006; Lohmann et al., 2013). A larger sampling within *Amphilophium* is necessary to further investigate the evolution of plastomes within the genus. Broader scale studies within tribe Bignonieae as a whole would certainly provide novel insights into the high diversity found in the structure, composition, and organization of plastomes in *Adenocalymma* (Fonseca and Lohmann, 2017), *Amphilophium* (this study), *Anemopaegma* (Firetti et al., 2017), and *Tanaecium* (Nazareno et al., 2015).

While the conservation of plastome structure and low levels of nucleotide diversity have been observed in several groups (Odintsova and Yurina, 2003; Wicke et al., 2011; Cai et al., 2015; Smith and Keeling, 2015; Reginato et al., 2016), our results show that plastomes may be variable within closely related lineages. Plastome rearrangements, differences in structure, size, gene content, and order were documented in many other angiosperm groups (Goulding et al., 1996; Chumley et al., 2006; Raubeson et al., 2007; Haberle et al., 2008; Wang et al., 2008; Guisinger et al., 2011; Dong et al., 2013; Weng et al., 2014; Zhu et al., 2016; Firetti et al., 2017; Fonseca and Lohmann, 2017; Park S. et al., 2018). Altogether, these results bring new insights into the evolution of plastomes, suggesting that plastomes may be highly conserved or highly variable in different plant groups. The analyses of complete Bignonieae plastomes indicate that genomes are variable at both the genus and species level within this tribe (Nazareno et al., 2015; Firetti et al., 2017; Fonseca and Lohmann, 2017).

### Variable Regions

The *rpoA, clpP, rps11, accD, rps12_5end/clpP, petD, trnN-GUU/ycf1, rpl32/trnL-UAG, rpl36, ycf1, rps18, matK/rps16*, and *ycf2* are identified as hypervariable loci at the species level within *Amphilophium* (Figure 3, 4). Furthermore, the *rpoA* gene shows the highest percentage of variable sites (25.6%) and the highest π value (0.06292) within *Amphilophium* plastomes. The *rpoA* gene does not show variability among members of Clade 5 though (i.e., *A. paniculatum, A. pilosum*, and *A. ecuadorense*), showing identical sequences in all taxa from this clade (Figure 3). Apart from encoding the subunits of one of the key chloroplast enzymes involved in tRNA and mRNA synthesis, the RNA polymerase type I (plastid-encoded polymerase, PEP), and the *rpo* genes (*rpoA, rpoB, rpoC1*, and *rpoC2*) are relatively rapidly evolving regions (Little and Hallick, 1988; Krawczyk and Sawicki, 2013). As a result, the *rpo* genes have been used in phylogeny reconstruction, with the *rpoC1* and *rpoB* genes representing DNA barcodes for land plants (Petersen and Seberg, 1997; Chase et al., 2007; Krawczyk and Sawicki, 2013). Similarly, to other angiosperm genera (Dugas et al., 2015), the *clpP* gene is also hypervariable within *Amphilophium* plastomes. More specifically, the *clpP* gene includes a loss of the *clpP* intron1 in *Inga* (Leguminosae), and accelerated rates of evolution in *clpP* in *Acacia* and *Inga* (Leguminosae) (Dugas et al., 2015), in Sileneae (Caryophyllaceae) (Sloan et al., 2014), and *Lamprocapnos spectabilis* (Papaveraceae) (Park S. et al., 2018). In terms of the number of variable sites (not considering sequence length), *ycf1* is the coding region with the highest number of variable sites within *Amphilophium* (346), followed by *rpoA* (281). The *ycf1* gene was also shown to represent the most variable region within *Anemopaegma* (Firetti et al., 2017), with 25.6% of variable sites. However, the *ycf1* gene shows only 5.5% of variable sites within *Amphilophium.* The relatively high divergence observed in the *ycf1, matK, rbcL*, and *accD* genes within *Amphilophium* plastomes is similar to that observed in plastomes of other angiosperms (Yukawa et al., 2006; Nie et al., 2012; Liu et al., 2013; Li and Zheng, 2018; Park S. et al., 2018; Zhao et al., 2018). Among the most divergent noncoding regions within *Amphilophium* plastomes, some were shown in previous studies to be highly variable and of high phylogenetic utility, i.e., *trnH-GUG/psbA, ndhF/rpl32, rpl32/trnL-UAG* (Shaw et al., 2005, 2007; Figure 3, 4). Three of the five introns and intergenic spacers selected as the most adequate markers for species level phylogenetics within the “*Adenocalymma-Neojobertia*” clade (Fonseca and Lohmann, 2017) are also variable within *Amphilophium* (i.e., *ndhA* intron, *clpP* intron 1, and *rpl32-trnL*). The remaining two markers (i.e., *petN/psbM* and *trnG* intron) selected for species-level phylogeny reconstruction within the “*Adenocalymma-Neojobertia*” clade (Fonseca and Lohmann, 2017), do not show significant sequence variation with *Amphilophium*, when compared to other regions (Figure 3, 4 and Supplementary Table S2).

### Signature of Positive Selection on Plastid Genes

Our study shows that among the 78 protein-coding genes within *Amphilophium*, 16 are significantly under positive selection (ω > 1) (i.e., *ycf1, ycf2, rpoA, accD, rps18, rps7, ycf4, clpP, rbcL, rpoC1, rps2, rpoC2, infA, atpA, rps8*, and *rps16*). Three of these genes (namely *ycf1, accD*, and *rbcL*) have been reported to be putatively under positive selection in Brassicaceae out of 10 genes identified with ω > 1 for the family (Hu et al., 2015). Within six species of *Ipomoea*, the genes *accD, cemA*, and *ycf2* were under positive selection (Park I. et al., 2018). Within eight *Anemopaegma*, on the other hand, four genes (i.e., *atpB, ndhA, petA*, and *psaB*) out of 70 protein-coding genes were shown to be under positive selection (Firetti et al., 2017). Positive selection on the *clpP* gene has been also observed in *Geranium* (Park et al., 2017), legume (Dugas et al., 2015), *Silene* (Erixon and Oxelman, 2008), and *Lamprocapnos* (Park S. et al., 2018) species. The chloroplast genes *ndhF* and *matK* also showed positive selection in previous studies. The *matK* gene is often used in phylogenetic studies (Carbonell-Caballero et al., 2015; Daniell et al., 2016) and showed to be positively selected in more than 30 plant groups, suggesting that this gene is subject to distinct ecological selective pressures (Chen and Xiao, 2010; Daniell et al., 2016). The positive selection signatures found on a high number of plastid genes within *Amphilophium*, suggest that these genes might be undergoing adaptative evolution in response to the environment (Kimura, 1989; Hu et al., 2015; Raman and Park, 2016; Ivanova et al., 2017). These results might be also associated with the remarkable morphological and ecological variation found among members of the genus. *Amphilophium* species show extremely diverse flower morphologies, occur in various environments, and show significant variation in diversification rates (Thode et al., 2019). Nonetheless, while plastid genes have been suggested to show signatures of positive selection (e.g., Erixon and Oxelman, 2008; Chen and Xiao, 2010; Carbonell-Caballero et al., 2015; Dugas et al., 2015; Hu et al., 2015; Daniell et al., 2016; Firetti et al., 2017; Park et al., 2017; Park I. et al., 2018), further studies that integrate field experiments, physiology, and molecular evolutionary biology are needed to understand this topic and the significance of adaptative evolution in plastid genes (Bock et al., 2014). Plastomes are shaped by the selective forces that act on the fundamental cellular functions that they code for and are, thus, expected to display signatures of the adaptive path undertaken by different plant species during evolution (Hu et al., 2015). Understanding the patterns of adaptation and divergence among the representatives of specific phylogenetic clades may provide important insights about the forces driving evolution (Wicke et al., 2014; Hu et al., 2015).

### SSRs in *Amphilophium* Plastomes

Single Sequence Repeats (SSRs) are repeats of 1–6 bp frequently observed in plastomes that are important markers for evolutionary studies, population genetics, and for the study of genome polymorphisms (Avise, 1994; Ebert and Peakall, 2009; Qi et al., 2016; Yu et al., 2017). In this study, the number of SSRs found within *Amphilophium* plastomes ranged from 44 to 57, while 42 SSRs are documented in *Anemopaegma prostratum*. These results are similar to the 36–47 SSRs documented previously for *Anemopaegma* plastomes (Firetti et al., 2017), but significantly lower than the 347 chloroplast SSRs found for *Tanaecium tetragonolobum* (Nazareno et al., 2015). In these two studies (Nazareno et al., 2015; Firetti et al., 2017), the SSRs were identified with a less stringent threshold than the one used here (i.e., seven to mononucleotide repeats, four to di- and three to, tri-, tetra-, penta-, and hexa-). As in *Anemopaegma* and *Tanaecium*, mononucleotide repeats are the most common SSRs found in noncoding regions of *Amphilophium* plastomes. Most SSRs contain A or T motifs, contributing to the overall plastome AT richness (Qian et al., 2013; Cauz-Santos et al., 2017; Park et al., 2017; Li and Zheng, 2018). The largest amount of SSRs is located in the LSC. These SSRs will be useful for future population genetic studies involving *Amphilophium* (Figure 5A–C). Dispersed repeats represent a major component of plastomes and influence genome structure in terms of genome size, genome recombination and rearrangements, and gene duplication (Cavalier-Smith, 2002; Nie et al., 2012). In this study, the number of repeats in *Amphilophium* plastomes found by REPuter range from 38 to 56, with 50 repeats being found in *Anemopaegma prostratum.* This finding was similar in *Tanaecium tetragonolobum*, which included 47 repeats (Nazareno et al., 2015), but different to eight other *Anemopaegma* species studied that showed between 88 and 169 dispersed repetitive sequences, the highest number documented within Lamiales to date (Firetti et al., 2017). Most repeat sequences within *Amphilophium* are 30–39 bp long, except from the repeats found in *A. carolinae, A. dolichoides*, and *A. steyermarkii* (Figure 5D). These three species show multiple repeats with 40–49 bp and the largest number of repeats >80 bp. Most dispersed repetitive sequences are found in noncoding regions (Figure 5F).

## Conclusion and Future Directions

The comparative analyses involving 11 *Amphilophium* plastomes and the plastome of *Anemopaegma prostratum* provided important new insights into Bignoniaceae plastome structure and evolution. Within *Amphilophium*, plastomes show different boundaries between the IR/SC regions, lengths, and number of duplicated genes in the IRs as well as high nucleotide variability and signature of positive selection. Our results show that plastomes may be highly variable, even at low taxonomic levels, indicating that differences in plastome structure, gene content, and nucleotide diversity vary among different plant groups. A larger sampling of taxa, including complete plastomes for a higher number of representatives of *Amphilophium* and other genera of tribe Bignonieae is necessary to further investigate the evolution of plastome structure in the genus and in the tribe as a whole.

## Author Contributions

Both authors designed the study, defined sampling and obtained samples, interpreted the results and co-wrote the manuscript. VT conducted the molecular work, assembled Illumina sequences, annotated plastomes, and performed analyses.

## Conflict of Interest Statement

The authors declare that the research was conducted in the absence of any commercial or financial relationships that could be construed as a potential conflict of interest.

## Footnotes

1 http://bitbucket.org/afinit/afin/

2 https://irscope.shinyapps.io/irapp/

3 http://wolfe.gen.tcd.ie/GenomeVx
