# Supplementary material for: Comparative Chloroplast Genomics at Low Taxonomic Levels: A Case Study Using Amphilophium (Bignonieae, Bignoniaceae)
Source: Front Plant Sci. 2019 Jun 19;10:796. doi: 10.3389/fpls.2019.00796 (PMC6594259; doi:10.3389/fpls.2019.00796)

Supplementary Material

Comparative chloroplast genomics at low taxonomic levels: A case study using *Amphilophium* (Bignonieae, Bignoniaceae)

Verônica A. Thode[1*] and Lúcia G. Lohmann[1*]

*** Correspondence:** *veronicathode@hotmail.com, llohmann@usp.br*

frontiers

SUPPLEMENTARY TABLES

Supplementary Table 1. Number of reads and average read depth for the assembled plastomes.

Species	Number of reads	Average read depth
Amphilophium carolinae (Lindl.) L. G. Lohmann	12,688,841	85.4
Amphilophium chocoensis (A. H. Gentry) L. G. Lohmann	14,885,219	100.1
Amphilophium cuneifolium (DC.) L. G. Lohmann	8,102,426	54.5
Amphilophium dolichoides (Cham.) L. G. Lohmann	23,885,903	248.0
Amphilophium dusenianum (Kraenzl.) L. G. Lohmann	9,027,451	60.7
Amphilophium ecuadorense A. H. Gentry	15,363,284	103.3
Amphilophium gnaphalanthum (A. Rich.) L. G. Lohmann	16,579,322	111.5
Amphilophium lactiflorum (Vahl) L. G. Lohmann	12,733,177	85.6
Amphilophium paniculatum (L.) Kunth	11,765,398	79.1
Amphilophium pilosum Standl.	13,946,760	93.8
Amphilophium steyermarkii (A. H. Gentry) L. G. Lohmann	10,369,861	69.8
**Outgroup**		
Anemopaegma prostratum DC.	15,876,937	106.8

Supplementary Table 2. Summary of the 78 protein-coding genes extracted from the plastomes, alignment length (bp), number of variable sites, and percentage of variable sites for the *Amphilophium* species aligned with *Anemopaegma prostratum* and for *Amphilophium* species only. Number of codon sites under positive selection detected using CODEML application in PAML with ω > 1 and Pr > 0.95.

Gene	Alignment length (bp) with *Ane. prostratum*	Number of codons	Variable sites with *Ane. prostratum*	% variable with *Ane. prostratum*	Positive selected sites P>95% (codeml-model 2-BEB)	Variable sites *Amphilophium* only	% variable *Amphilophium* only
**accD**	2676	892	227	8.48	12	198	7.40
**atpA**	1518	506	42	2.77	1	35	2.31
**atpB**	1497	499	28	1.87	0	27	1.80
**atpE**	402	134	9	2.24	0	9	2.24
**atpF**	555	185	20	3.60	0	19	3.42
**atpH**	246	82	4	1.63	0	4	1.63
**atpI**	744	248	19	2.55	0	16	2.15
**ccsA**	972	324	48	4.94	0	42	4.32
**cemA**	687	229	20	2.91	0	18	2.62
**clpP**	597	199	91	15.24	5	78	13.07
**infA**	282	94	24	8.51	2	14	4.96
**matK**	1536	512	87	5.66	0	80	5.21
**ndhA**	1092	364	44	4.03	0	39	3.57
**ndhB**	1533	511	9	0.59	0	7	0.46
**ndhC**	363	121	5	1.38	0	5	1.38
**ndhD**	1503	501	72	4.79	0	61	4.06
**ndhE**	306	102	4	1.31	0	4	1.31
**ndhF**	2232	744	133	5.96	0	114	5.11
**ndhG**	531	177	24	4.52	0	21	3.95
**ndhH**	1182	394	63	5.33	0	56	4.74
**ndhI**	507	169	18	3.55	0	16	3.16
**ndhJ**	477	159	13	2.73	0	13	2.73
**ndhK**	678	226	12	1.77	0	8	1.18
**petA**	969	323	32	3.30	0	29	2.99
**petB**	648	216	12	1.85	0	10	1.54
**petD**	483	161	12	2.48	0	10	2.07
**petG**	114	38	2	1.75	0	2	1.75
**petL**	96	32	4	4.17	0	3	3.13
**petN**	90	30	4	4.44	0	3	3.33
**psaA**	2253	751	38	1.69	0	32	1.42
**psaB**	2205	735	45	2.04	0	32	1.45
**psaC**	246	82	9	3.66	0	9	3.66
**psaI**	111	37	3	2.70	0	3	2.70
**psaJ**	135	45	4	2.96	0	4	2.96
**psbA**	1056	352	24	2.27	0	21	1.99
**psbB**	1527	509	49	3.21	0	38	2.49
**psbC**	1422	474	26	1.83	0	25	1.76
**psbD**	1062	354	14	1.32	0	12	1.13
**psbE**	252	84	5	1.98	0	5	1.98
**psbF**	120	40	3	2.50	0	3	2.50
**psbH**	222	74	4	1.80	0	4	1.80
**psbI**	111	37	5	4.50	0	5	4.50
**psbJ**	123	41	2	1.63	0	2	1.63
**psbK**	180	60	4	2.22	0	3	1.67
**psbL**	117	39	1	0.85	0	1	0.85
**psbM**	105	35	3	2.86	0	3	2.86
**psbN**	132	44	3	2.27	0	2	1.52
**psbT**	102	34	2	1.96	0	1	0.98
**psbZ**	189	63	4	2.12	0	2	1.06

(cont.)

Supplementary Table 2. Cont.

Gene	Alignment length (bp) with *Ane. prostratum*	Number of codons	Variable sites with *Ane. prostratum*	% variable with *Ane. prostratum*	Positive selected sites P>95% (codeml-model 2-BEB)	Variable sites *Amphilophium* only	% variable *Amphilophium* only
**rbcL**	1434	478	38	2.65	5	31	2.16
**rpl14**	369	123	6	1.63	0	6	1.63
**rpl16**	408	136	10	2.45	0	7	1.72
**rpl2**	825	275	7	0.85	0	7	0.85
**rpl20**	432	144	26	6.02	0	23	5.32
**rpl22**	474	158	16	3.38	0	13	2.74
**rpl23**	306	102	8	2.61	0	1	0.33
**rpl32**	177	59	11	6.21	0	10	5.65
**rpl33**	201	67	6	2.99	0	6	2.99
**rpl36**	114	38	14	12.28	0	10	8.77
**rpoA**	1116	372	405	36.29	15	281	25.18
**rpoB**	3228	1076	162	5.02	0	112	3.47
**rpoC1**	2046	682	94	4.59	4	70	3.42
**rpoC2**	4023	1341	234	5.82	2	159	3.95
**rps11**	489	163	65	13.29	0	55	11.25
**rps12**	378	126	5	1.32	0	5	1.32
**rps14**	303	101	7	2.31	0	6	1.98
**rps15**	222	74	2	0.90	0	2	0.90
**rps16**	240	80	19	7.92	1	11	4.58
**rps18**	456	152	57	12.50	11	47	10.31
**rps19**	279	93	3	1.08	0	3	1.08
**rps2**	714	238	60	8.40	4	56	7.84
**rps3**	663	221	30	4.52	0	28	4.22
**rps4**	642	214	41	6.39	0	39	6.07
**rps7**	462	154	13	2.81	11	12	2.60
**rps8**	405	135	19	4.69	1	13	3.21
**ycf1**	6279	2093	488	7.77	31	346	5.51
**ycf2**	6579	2193	321	4.88	25	270	4.10
**ycf3**	507	169	6	1.18	0	5	0.99
**ycf4**	597	199	55	9.21	8	33	5.53
Total	70554	23518	3658		138	2868	

Supplementary Table 3. Results from the distribution of SSRs in all plastomes of the *Amphilophium* species studied here and *Anemopaegma prostratum*.

Species	SSRs total	Unit size						Total per region			SSRs% per region			A/T repeats	% of A/T repeats	A repearts	T repearts	Total per region			SSR% per region		
		mono	di	tri	tetra	penta	hexa	LSC	IR	SSC	LSC	IR	SSC					exon	intron	IGS	exon	intron	IGS
A. carolinae	52	31	4	4	6	5	2	37	11	4	71.2	21.2	7.7	29	55.8	12	17	9	8	35	17.3	15.4	67.3
A. chocoensis	47	30	3	7	5	1	1	39	4	4	83.0	8.5	8.5	30	63.8	20	10	11	8	28	23.4	17.0	59.6
A. cuneifolium	50	33	3	7	5	2	0	42	4	4	84.0	8.0	8.0	33	66.0	21	12	10	8	32	20.0	16.0	64.0
A. dolichoides	50	29	4	5	6	4	2	36	11	3	72.0	22.0	6.0	27	54.0	11	16	9	6	41	18.0	12.0	82.0
A. dusenianum	57	39	3	7	7	1	0	45	7	5	78.9	12.3	8.8	37	64.9	16	21	13	10	34	22.8	17.5	59.6
A. ecuadorense	49	32	3	7	4	2	1	40	8	1	81.6	16.3	2.0	30	61.2	13	17	9	10	30	18.4	20.4	61.2
A. gnatophalantum	55	38	2	6	9	0	0	46	6	3	83.6	10.9	5.5	38	69.1	22	16	13	9	33	23.6	16.4	60.0
A. lactiflorum	54	36	2	6	9	1	0	44	6	4	81.5	11.1	7.4	34	63.0	20	14	13	10	31	24.1	18.5	57.4
A. paniculatum	44	30	3	6	4	1	0	38	5	1	86.4	11.4	2.3	29	65.9	14	15	7	8	29	15.9	18.2	65.9
A. pilosum	48	33	3	6	4	1	1	40	5	3	83.3	10.4	6.3	32	66.7	16	16	7	10	31	14.6	20.8	64.6
A. steyermarkii	46	34	2	3	6	1	0	37	5	4	80.4	10.9	8.7	32	69.6	14	18	11	8	27	23.9	17.4	58.7
Ane. prostratum	42	27	3	6	3	1	2	29	10	3	69.0	23.8	7.1	26	61.9	13	13	10	4	28	23.8	9.5	66.7

Supplementary Table 4. Results from the REPuter analysis of distribution of repeat sequences in the plastomes of the *Amphilophium* species studied here and *Anemopaegma prostratum*. F = forward, P = palindrome, and R reverse repeats.

Species	Total	Total repeats per region			Repeat type			Maximum size	Number of repeats per size						Number of repeats per region		
		LSC	IR	SSC	F	P	R		30-39bp	40-49bp	50-59bp	60-69bp	70-79bp	>80bp	exon	IGS	intron
A. carolinae	50	23	27	0	49	1	0	141	3	21	5	11	4	6	29	20	1
A. chocoensis	49	33	16	0	42	6	1	84	23	17	2	5	1	1	23	18	8
A. cuneifolium	50	17	33	0	47	3	0	91	15	15	7	7	1	5	20	27	3
A. dolichoides	50	8	42	0	49	1	0	150	1	18	13	6	4	8	24	26	0
A. dusenianum	56	17	39	0	49	4	3	115	29	15	4	4	1	3	33	18	5
A. ecuadorense	53	11	42	0	50	3	0	95	27	14	6	3	1	2	19	32	2
A. gnatophalantum	42	20	22	0	37	5	0	82	25	10	3	2	1	1	20	18	4
A. lactiflorum	38	18	20	0	33	5	0	88	25	8	1	1	2	1	18	14	6
A. paniculatum	50	7	43	0	48	2	0	50	20	18	5	3	4	0	12	36	2
A. pilosum	50	15	35	0	47	3	0	109	24	13	3	4	2	4	20	30	0
A. steyermarkii	50	13	37	0	48	2	0	150	8	18	5	9	1	9	31	18	1
Ane. prostratum	50	3	47	0	49	1	0	165	0	13	9	14	7	7	24	26	0

Supplementary Table 5. Results from the REPuter analysis of distribution of repeat sequences in the plastomes of the *Amphilophium* species studied here and *Anemopaegma prostratum*.

**Species/ Location of repeat 1**	***A. paniculatum***	***A. pilosum***	***A. ecuadorense***	***A. dusenianum***	***A. gnatophalantum***	***A. lactiflorum***	***A. steyermakii***	***A. dolichoides***	***A. carolinae***	***A. chocoensis***	***A. cuneifolium***	***Ane. prostratum***
accD	2	2	3	4	2	5	9	6	10	7	3	
rbcL/accD	1	4	1	1	1	1	1	1	1	2	1	
ycf1	1	2	3	12	4	4	13	11	12	6	11	14
ycf2	7	11	9	8	7	6	6	7	7	5	3	8
rps12/trnV-GAC	1	1	1	1	1	1	1	1	1	1	1	
trnN-GUU/ycf1	27	12	8	1	1	1	1	1	3		1	
ycf3	2	2	2	3	4	4	1		1	3	2	
psbT/psbN	1	1	1	1	1	1	1			1	1	1
rps11	1	1	2	5	3	1	1			1	1	1
rpl23/trnI-CAU	6	7	19		8	4	8			3	17	
rps18	1	1	1	3	2		1			1	1	1
psbI/trnS-GCU		1	1	2	2	2				2	1	
psaB		1	1	1	1	1				1		
atpF/atpH					1	1				1		
psbC/trnS-UGA					1	1				1		
rps11/rpl36							4	10	5			
rrn4.5/rrn5					1	1				1		
trnI-CAU/ycf2							1	11	4			
clpP										5	1	
clpP/psbB										1	1	
petN/psbM										1		
psaA/ycf3				1								
psaJ/rpl33			1							2	1	
rpl2				11		2						
rpl33/rps18								1	5			
rpl36/infA								1	1			
rpoA										2	1	
rpoA/rps11							1			1	2	
trnL-UAA/trnF-GAA										1	1	
atpB/rbcL					1							
psaI/ycf4		4										1
psbM/trnD-GUC						1						
rpl22							1					
rpoC1				2								
rps15						1						
ycf4					1							
rpl2/rpl23												2
rps12												1
trnI-AAU/ndhB												21
**Total**	**50**	**50**	**53**	**56**	**42**	**38**	**50**	**50**	**50**	**49**	**50**	**27**

SUPPLEMENTARY FIGURE

Supplementary Figure 1. Gene map of *A. gnatophalantum* and *A. steyermarkii*, the smallest and largest *Amphilophium* plastomes sequenced in this study. Genes drawn inside the circle are transcribed clockwise, and those outside are transcribed counterclockwise. Genes belonging to different functional groups are colored following the legend. The darker and lighter gray in the inner circle correspond to GC content and AT content, respectively.

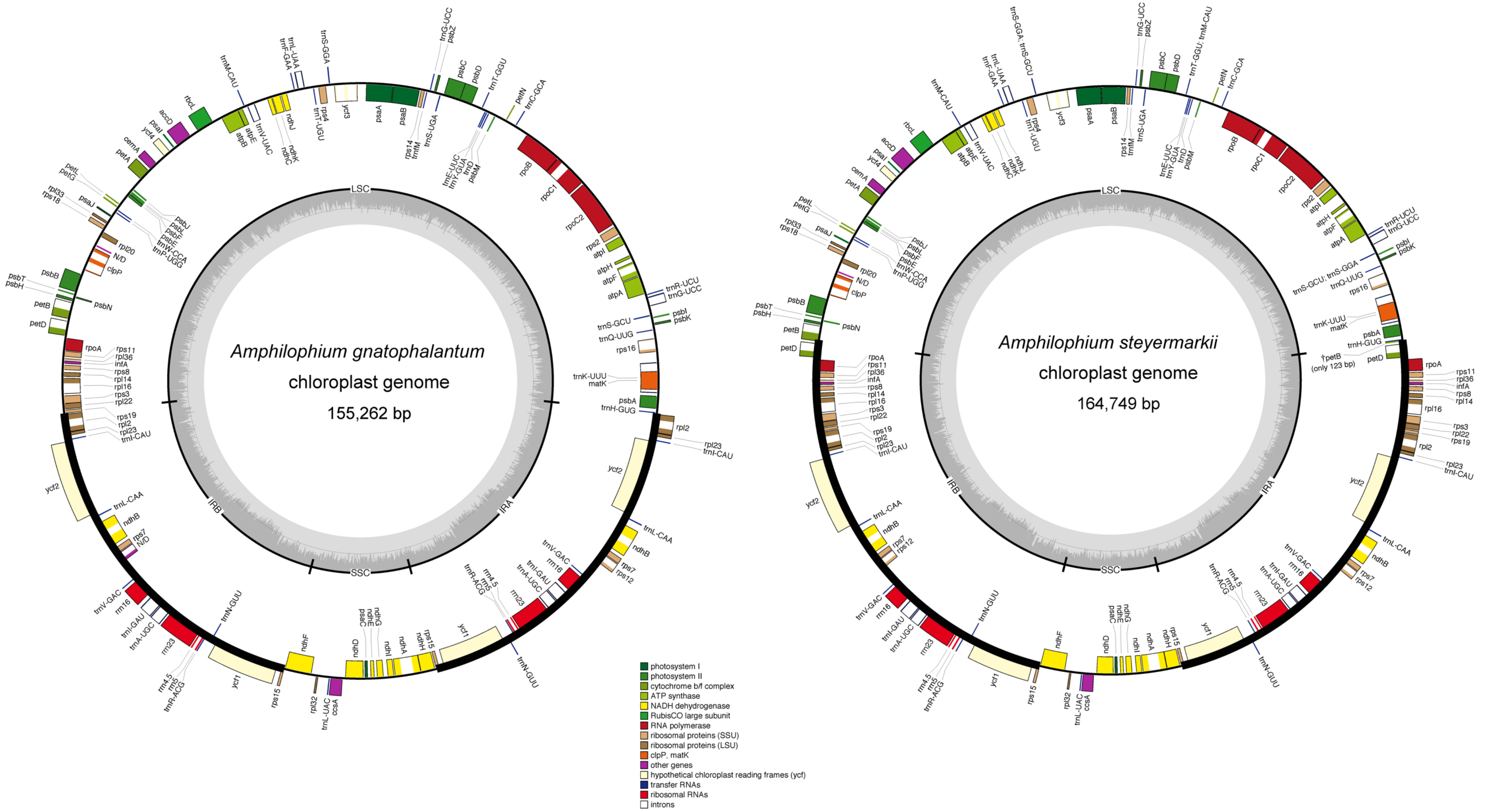

Supplementary Figure 2. Comparison of *Amphilophium* and *Anemopaegma prostratum* cp genomes. **A**. Large Single Copy (LSC), Inverted Repeat (IR), and Small Single Copy regions lengths in base pairs (bp). **B**. Coding and noncoding regions lengths in bp.

A

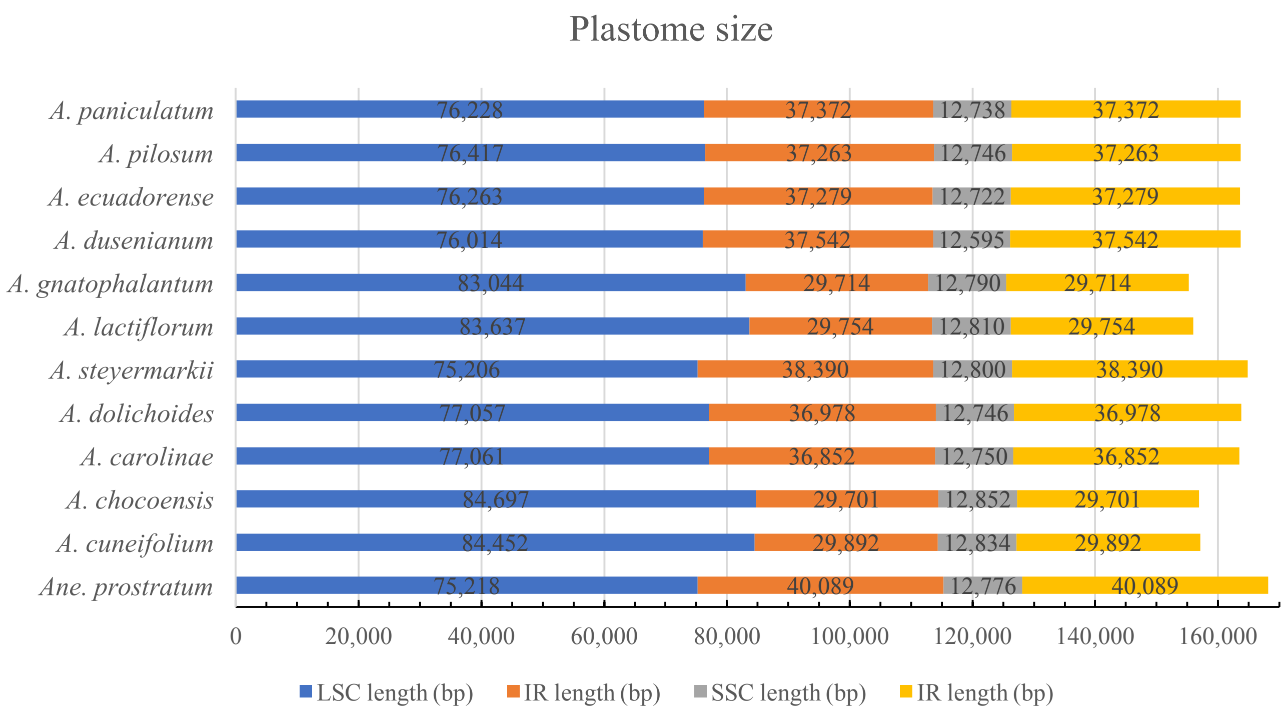

B

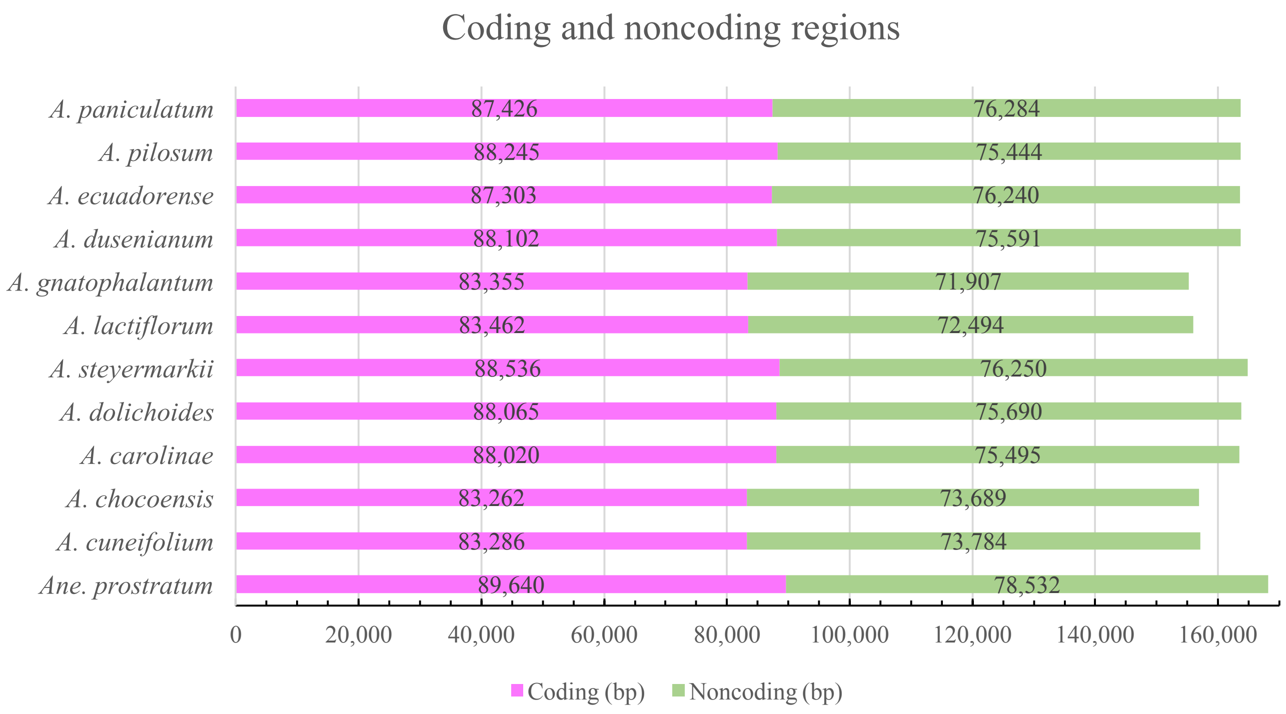

Supplementary Figure 3. Synteny and rearrangements detected in the plastomes from the 11 sequenced *Amphilophium* species, *Adenocalymma peregrinum*, *Anemopaegma arvense*, and *Tanaecium tetragonolobum* using the Mauve multiple-genome alignment program. Yellow blocks showing thr *ycf2* region inversion in the cp genomes of the *Anemopaegma* species and the blue blocks showing the inversion within the *ycf1* gene in *Tanaecium tetragonolobium*.

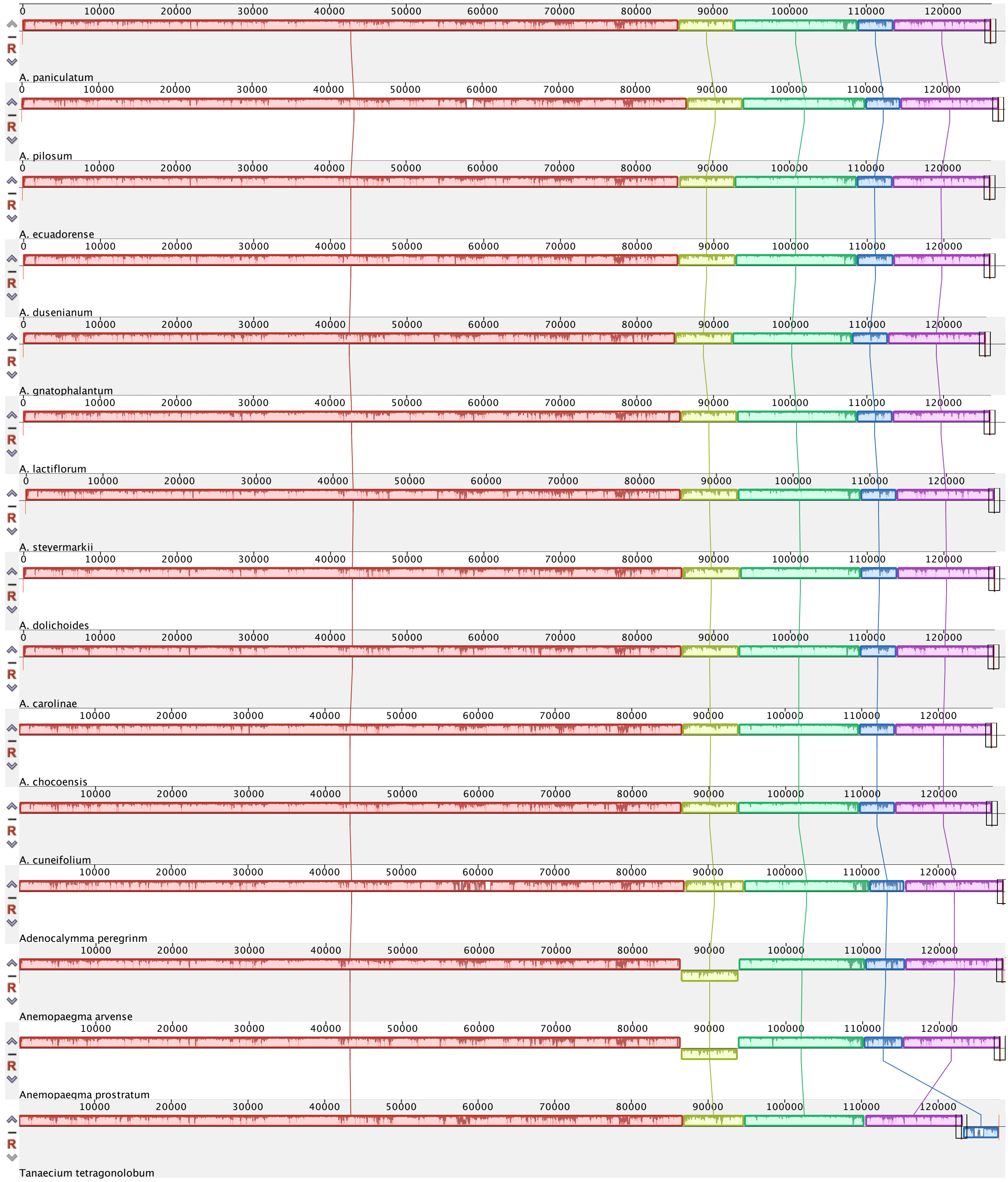

Supplement: Supplementary file 1 [file Data_Sheet_2.PDF]
